# Development of Medicines for Rare Pediatric Diseases

**DOI:** 10.3390/ph16040513

**Published:** 2023-03-30

**Authors:** Danilo Marimpietri, Guendalina Zuccari

**Affiliations:** 1 Cell Factory, IRCCS Istituto Giannina Gaslini, Via Gerolamo Gaslini 5, 16147 Genoa, Italy; danilomarimpietri@gaslini.org; 2 Department of Pharmacy (DiFAR), University of Genoa, Viale Benedetto XV 3, 16132 Genoa, Italy

To date, approximately 7000 rare diseases exist, affecting between 6% and 8% of the global population and >30 million people in the European Union. Symptoms often occur early at birth or during childhood and may progressively increase, becoming chronic or relapsing, leading to life-threatening conditions. In fact, 75% of rare diseases are known to affect children, and about 3 out of 10 of these children die before reaching 5 years of age. Furthermore, only 5% of rare diseases have effective treatment options, which leaves 95% of rare diseases without any approved medicines. Therefore, incentivizing medicine development is crucial for this subset of the population, which is disproportionately affected by rare diseases. Since both the European Orphan Regulation on Medicinal Product, first, and the European Paediatric Regulation, later, came into force, the development of medicines suitable for pediatrics has consistently increased, but much more needs to be done. To date, the medical and scientific knowledge on rare diseases remains limited, and the absence of approved treatment options has led physicians and pharmacists to the harmful off-label use of medicines.

Our call for papers for this Special Issue received great interest from a broad range of researchers mainly involved in the field of pharmaceutical technology, hospital pharmacy, clinical and basic medical research. A total of 85 authors from all over the world (Italy, Spain, Portugal, Taiwan, Belgium, UK, Austria, Germany, Nigeria, USA) contributed to nine research papers and three reviews.

Since the most available marketed pharmaceutical products are not intended for pediatrics, Carvalho et al. and Zuccari et al. underlined the role of the pharmacist in compounding tailor-made formulations suitable for children [1,2]. Particularly, Carvalho et al. rose the issue of the relevance of compounding a customized pharmaceutical product on medication adherence. Indeed, when a medicinal product is prepared in a pharmacy, patients are more involved, more confident in the drug efficacy, and more in contact with the pharmacist, paving the way for the role of pharmacist in educating patients. Despite these benefits, studies addressing the contribution of compounding and dispensing “the best-fit formulation” on patient compliance are lacking in the literature. In the review, the authors propose a methodology of Patient-Centric Compounding Design (PCCD) to systematize the practice of preparing tailored medicines [1].

Zuccari et al. reviewed the current state-of-the-art mini tablet preparation, highlighting the huge potential of this dosage form for the pediatric population. The authors underscore the advantages of solid preparations on traditional liquid pediatric formulations, which may contain additives such as flavors, sweeteners, preservatives, surfactants, and cosolvents, potentially harmful for children. Moreover, the experience of the hospital pharmacy at G. Gaslini Children’s hospital in daily medicine manipulation is reported. The authors concluded that the development of oral pediatric dosage form is still challenging despite the support of regulatory authorities, and that 3D printed mini tablets may be a promising option. Indeed, they could reduce the number of ingredients and at the same time be flexible and customizable [2].

The third review is focused on the use of nanometric delivery systems in the treatment of retinoblastoma, a rare pediatric cancer of the retina. Currently, conventional therapeutic approaches are based on radiotherapy and systemic chemotherapy combined with aggressive focal therapies, but they are strongly associated to the onset of broad side effects. To reduce undesired reactions and improve efficacy of conventional therapies, nanomedicine may represent a valid adjuvant. In fact, the main issue related to ocular therapy is the ability of a drug to pass the anatomical barriers such as cornea, conjunctiva, sclera and blood–retinal barrier, and this problem could be successfully overcome with the administration of nanoparticles [3].

As further evidence of the possible applications of nanotechnology in the field of drug administration, three papers dealing with the preparations and characterization of nanoparticles are included in this Special Issue. Curiously, the nanoparticles were prepared by the same method: the nanoprecipitation method–dropping technique. Indeed, compared to other techniques used to produce polymeric NPs, such as the commonly used emulsification and solvent evaporation method, nanoprecipitation is an easy, scalable and reproducible method that avoids the loss of drugs during preparation. It is worth mentioning that when developing pediatric medicines, the formulator should bear in mind that the number of ingredients must be as low as possible to avoid potentially toxic or unsuitable excipients for children. To comply with safety requirements, the authors did not use surfactants to stabilize the colloidal dispersion [4,5,6]. Nieto González et al. developed nanoparticles based on chitosan and cellulose acetate phthalate containing captopril (a hydrophilic drug) for the treatment of hypertension, heart failure and diabetic nephropathy in pediatric patients [4]. Nanoprecipitation is mainly employed for the encapsulation and solubilization of hydrophobic drugs, thus Zuccari et al. first prepared nanoparticles made of a water-soluble cationic copolymer (P5) obtained by copolymerizing the laboratory-made monomer 4-ammoniumbuthylstyrene hydrochloride with di-methyl-acrylamide as an uncharged diluent, and loaded with fenretinide, a lipophilic retinoid, known to be active against neuroblastoma [5]. Second, the authors employed another positive copolymer (P7) to enhance the antitumor activity of a pyrazole derivative (CB1H) poorly soluble in water [6].

Another formulative study aimed at ameliorating the palatability of a bitter drug (ranitidine) was performed by Ogbonna et al. The study applies a patient centric design process which includes the definition of a target product profile (TPP). The developed solution consisted of a mixture of sodium saccharine and aspartame and 0.1% sodium chloride, which allowed a higher masking effectiveness than simple syrup. The final oral formulation matched the TPP in all dimensions, namely composition suitable for children, preparation and handling adapted to hospital pharmaceutical compounding and adequate stability and quality [7].

The contribution from Raffaghello et al. regarded the evaluation of the therapeutic effectiveness of a selective P2X7 purinoreceptor antagonist, A438079, for the treatment of limb–girdle muscular dystrophy R3, a rare genetic disorder affecting the limb proximal muscles caused by mutations in the α-sarcoglycan gene. In vivo experiments on α-sarcoglycan null mice demonstrated the ability of A438079 in counteracting the progression of the dystrophic phenotype and reducing the inflammatory response. This study paves the way to P2X7 antagonism via selective inhibitors as a valid immunosuppressant strategy aimed to dampen the basal immune-mediated damage and to favor a better engraftment of gene–cell therapies [8].

The remaining papers originate from clinical studies. Recently, the emergence of the spread of *Candida auris* among preterm neonates has been declared. De Rose et al. reported the requirement in neonates of higher doses of micafungin than that of adults to treat systemic candidiasis for increased plasma clearance. The authors assessed that the drug doses ranging from 8 to 15 mg/kg/day administered to 53 neonates and infants are safe and effective [9].

Wu et al. investigated the addition of antithymocyte globulin (ATG) in haploidentical hematopoietic stem cell transplantation using post-transplant cyclophosphamide (PTCy) for graft-versus-host disease (GVHD) prophylaxis in children. To date, the PTCy-based regimens for GVHD reduction were reported only for adults. The authors demonstrated that ATG plus PTCy is an effective strategy for GVHD and is feasible in children with high-risk malignancies. However, the main limitations of this study were the small cohort of patients and the short follow-up, and larger cohorts of patients are needed to validate the results and optimize the protocol [10].

Stachanow et al. developed a modeling and simulation framework to monitor 17α-hydroxyprogesterone (17-OHP) as biomarker in cortisol replacement therapy for patients affected with congenital adrenal hyperplasia. The less invasive dried blood spot (DBS) sampling is an advantageous alternative to traditional plasma sampling, especially in pediatric patients. However, target concentrations for 17-OHP are unknown using DBS. The framework included a PK/PD model which successfully linked DBS 17-OHP to plasma cortisol concentrations in pediatric CAH patients. By leveraging this framework, the authors were able to derive a plausible target morning concentration range for the biomarker 17-OHP in the range of 2–8 nmol/L, which is applicable for both venous and capillary DBS samples [11].

Conducting clinical trials in pediatric or rare diseases is often fraught with risk and uncertainty due to the heterogeneity and the small number of patients. This makes individual treatment trials (ITTs), also known as n-of-1 trials, an excellent alternative. ITTs rely on the use of a treatment without scientifically proven efficacy or outside indications when established treatment methods are no longer helpful. Therefore, ITTs can be extremely useful for investigating new effects of care in diseases with unsatisfactory therapies [12].

In summary, this special issue highlights the challenges of treating the pediatric population, from the preparation of appropriate medicines to the need for new drugs and new clinical regimes. We sincerely thank all the authors for their valuable contributions, hoping that this Special Issue will promote pediatric disease research in the scientific community.

## Data Availability

Data sharing not applicable.
